# Hepatitis C core antigen test as an alternative for diagnosing HCV infection: mathematical model and cost-effectiveness analysis

**DOI:** 10.7717/peerj.11895

**Published:** 2021-09-10

**Authors:** Maryam Sadeghimehr, Barbara Bertisch, Francesco Negro, Maia Butsashvili, Sonjelle Shilton, Irina Tskhomelidze, Maia Tsereteli, Olivia Keiser, Janne Estill

**Affiliations:** 1 Institute of Global Health, University of Geneva, Geneva, Switzerland; 2 Checkin Helvetiaplatz, Zürich, Switzerland; 3 Divisions of Gastroenterology and Hepatology and of Clinical Pathology, Geneva University Hospitals, Geneva, Switzerland; 4 Clinic Neolab, Tbilisi, Georgia; 5 FIND, Geneva, Switzerland; 6 TEPHINET for Georgia Hepatitis C Elimination Program, I. Javakhishvili Tbilisi State University, Tbilisi, Georgia; 7 Department of HIV/AIDS, Hepatitis, STI and TB, National Center for Disease Control and Public Health, Tbilisi, Georgia; 8 Institute of Mathematical Statistics and Actuarial Science, University of Bern, Bern, Switzerland

**Keywords:** HCV, Hepatitis C, PCR, Polymerase chain reaction, Antigen, Diagnostic test, Mathematical modeling, Progression model, Screening strategies, Country of Georgia

## Abstract

**Background:**

The cost and complexity of the polymerase chain reaction (PCR) test are barriers to diagnosis and treatment of hepatitis C virus (HCV) infection. We investigated the cost-effectiveness of testing strategies using antigen instead of PCR testing.

**Methods:**

We developed a mathematical model for HCV to estimate the number of diagnoses and cases of liver disease. We compared the following testing strategies: antibody test followed by PCR in case of positive antibody (baseline strategy); antibody test followed by HCV-antigen test (antibody-antigen); antigen test alone; PCR test alone. We conducted cost-effectiveness analyses considering either the costs of HCV testing of infected and uninfected individuals alone (A1), HCV testing and liver-related complications (A2), or all costs including HCV treatment (A3). The model was parameterized for the country of Georgia. We conducted several sensitivity analyses.

**Results:**

The baseline scenario could detect 89% of infected individuals. Antibody-antigen detected 86% and antigen alone 88% of infected individuals. PCR testing alone detected 91% of the infected individuals: the remaining 9% either died or spontaneously recovered before testing. In analysis A1, the baseline strategy was not essentially more expensive than antibody-antigen. In analysis A2, strategies using PCR became cheaper than antigen-based strategies. In analysis A3, antibody-antigen was again the cheapest strategy, followed by the baseline strategy, and PCR testing alone.

**Conclusions:**

Antigen testing, either following a positive antibody test or alone, performed almost as well as the current practice of HCV testing. The cost-effectiveness of these strategies depends on the inclusion of treatment costs.

## Introduction

Hepatitis C virus (HCV) is a major cause of liver disease and liver-related mortality (Pawlotsky et al., 2018). The World Health Organization (WHO) estimates that 71 million people worldwide are chronically infected with hepatitis C, and 400,000 people die from HCV every year, mostly due to cirrhosis and hepatocellular carcinoma. However, the majority of the HCV infected individuals are not aware of their infection (WHO, 2021). Effective hepatitis testing strategies and tools are needed to achieve the WHO target of eliminating HCV as a major public health threat by 2030 (World Health Organization, 2016).

Since 2014, Direct Acting Antivirals (DAA) form the standard HCV treatment. For successful DAA treatments, tests are needed to diagnose the infection and confirm the clearance of viral replication (Pawlotsky et al., 2018; Tillmann, 2014). Two types of tests are usually applied: serological assays that detect antibodies to HCV, and nucleic acid tests that detect HCV RNA genomes to confirm active infection (Tillmann, 2014; Gretch, 2000). The most commonly used testing protocol is to first use an antibody test, and if the result is positive, check the presence of the virus with a nucleic acid test (usually a polymerase chain reaction test, PCR) (Tillmann, 2014; Gretch, 2000). The sensitivity and specificity of PCR tests are high (Gretch, 2000). PCR testing requires time and trained laboratory personnel, which increases the costs. The cost of PCR is an important barrier for comprehensive testing, especially in low- and middle-income countries.

HCV-antigen test is a serological assay that directly detects a viral protein, giving a positive result as soon as the virus component is present. The test can be done on the same platform as the antibody test (Tillmann, 2014), is cheaper (Cresswell et al., 2015) and requires less special training than PCR testing. While antigen tests have a specificity of up to 100%, viral loads below 3,000 IU/ml may not be detected (Tillmann, 2014; Bertisch et al., 2020).

With a limited budget, replacing PCR by antigen testing could increase testing coverage, but people with very low viral loads may be missed. Using the country of Georgia as an example, we aimed to study the cost-effectiveness of different testing strategies using a mathematical model.

## Materials and Methods

### Model structure and inputs

**Figure 1:**
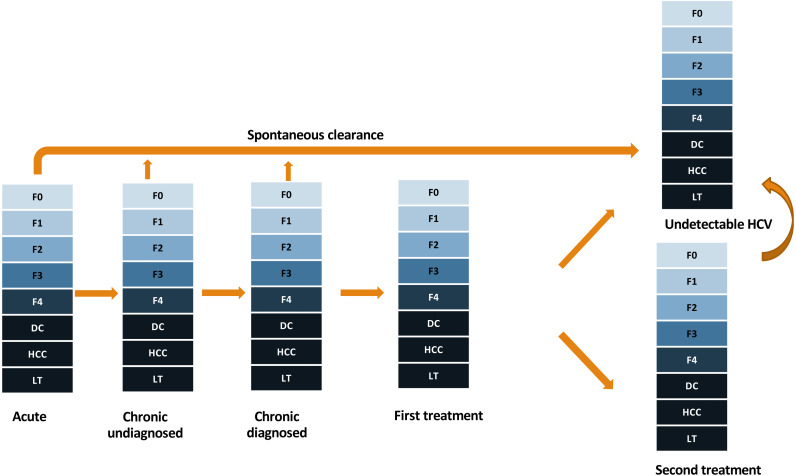
Structure of the simulation model. Individuals can progress vertically based on liver disease, and horizontally through the hepatitis C virus (HCV) infection and cascade of care. First and second treatments contain the treatment episode itself and, in case of treatment failure, the time after ending therapy. Death can occur at any state (not shown in the graph for simplicity). F0-F4, stages of fibrosis according to the METAVIR scoring system; DC, decompensated cirrhosis; HCC, hepatocellular carcinoma; LT, Liver transplantation.

We developed a mathematical model for HCV disease progression, similar to a previously published model (Sadeghimehr et al., 2019). We simulated cohorts of patients from infection until death. The progression of HCV is represented by a directed acyclic graph of health states. In each state, the model samples when and to which state the patient will move next. The process is repeated until the patient reaches a terminal state (death). The patients progress along the stages of liver disease, course of HCV infection and cascade of care (Fig. 1). The liver disease stages are represented by the METAVIR scoring system (F0-F4), followed by decompensated cirrhosis (DC), hepatocellular carcinoma (HCC) and liver transplantation (LT). At the beginning of the simulation, patients are assigned the following characteristics: age at infection, year of birth, gender, HIV co-infection, level of alcohol consumption, and duration of intravenous drug use (IDU).

Many studies have shown that hepatitis C viral load is relatively stable in untreated patients with chronic infection (Gretch, 2000; Nguyen et al., 1996). We therefore assumed that viral loads remain approximately constant in untreated individuals. We used the viral load distribution among patients in the Swiss Hepatitis C Cohort Study (Bertisch et al., 2020) and assigned each patient a baseline viral load. Viral load values at the time of HCV testing were sampled from a log-normal distribution around the baseline viral load. We denote viral loads below 3,000 IU/ml as very low viral loads (VLVL) (Tillmann, 2014; Bertisch et al., 2020).

We considered the following testing strategies: HCV-antibody followed by PCR testing in case of a positive antibody test (baseline strategy); HCV-antibody followed by HCV-antigen testing in case of a positive antibody test (antibody-antigen strategy); HCV-antigen test alone; and PCR test alone. In all strategies, a second test (either PCR or antigen, whichever was used to confirm the diagnosis) was taken 12 weeks after treatment completion to confirm sustained virologic response (SVR). We assumed that all individuals were tested for HCV once during the years 2015–2018. In case of a negative test result, the individual was not retested.

We assumed that the sensitivity of the antibody test increases exponentially during the first year of the infection and stabilizes at 99% thereafter (Thomson et al., 2009; Tang et al., 2017). The sensitivity of the antigen test was assumed to be 33.0% for patients with VLVL, and 98.2% for everyone else (Bertisch et al., 2020). The PCR test was assumed to be 100% sensitive. The expected numbers of tests among HCV uninfected people were calculated from the HCV prevalence in the target population. We assumed 100% specificity for HCV-antigen and PCR tests. We assumed that all detected patients are treated with DAAs, and 98% of the treated patients achieve SVR (Pawlotsky et al., 2018).

We parameterized the model for Georgia, one of the first countries that aimed to eliminate HCV. Enlarged-scale HCV screening began in January 2015, and the elimination programme (National Hepatitis C Virus Elimination Progress Report, 2018) was launched in April 2015. Screening services continue to be provided free of charge in various settings. As of June 30, 2018, a total of 1,175,291 HCV screening tests had been done and 1,125,808 persons registered in the elimination programme, of whom 93,181 (8.3%) were positive for HCV antibody. Currently in Georgia patients without documented HCV serological status first undergo anti-HCV antibody testing. Patients with positive anti-HCV antibodies undergo PCR testing, or since December 2017 alternatively core antigen testing (Ministry of Health of Georgia, 2020). Table 1, Table S1 and Figs. S1–S2 present the baseline characteristics of the simulated individuals, the model’s parameters and assumptions. We assumed that HCV viral loads were not independently associated with fibrosis progression rates (Heller & Seeff, 2005). The simulated population included patients infected before 2019 who had not cleared the virus spontaneously or been treated before 2015, and had not been diagnosed by 2015.

**Table 1:** Baseline characteristics of the simulated patients.

Characteristics	**Active IDU**	**Non-IDU**	**Source**
Alcohol consumption Abstinent Moderate (on average 20–40 g per day) Excessive (on average >40 g per day)	37% 37% 26%	37% 37% 26%	Butsashvili (2016), assumption
Gender Female Male	0.8% 99.2%	47.2% 52.8%	National Hepatitis C Virus Elimination Progress Report (2018), Butsashvili (2016), Stvilia et al. (2006)
HIV co-infected	2.3%	0.2%	UNAIDS (2018)
HCV prevalence	66.2%	5.4%	Strategic plan for the elimination of Hepatitis C Virus in Georgia (2020), Jülicher & Galli (2018)

**Notes.**

IDU injection drug user

### Model outcomes

We estimated the number of diagnoses for each testing strategy and compared the number of people who experienced severe liver disease (F3), cirrhosis (F4), DC, HCC, and liver-related death.

We also compared the cost-effectiveness of the testing alternatives, conducting three analyses with different assumptions regarding costs. In analysis A1, we only considered the direct costs of the HCV tests, including also testing the HCV uninfected individuals not explicitly simulated. In analysis A2, we added the costs of HCV-associated consultations with clinical assessment, complete blood count and alanine aminotransferase test (Ministry of Health of Georgia, 2020) and the lifetime costs associated with liver disease. This analysis takes the perspective of the health care payer in situations like in the country of Georgia where treatment costs are covered by external donors. In analysis A3, we included all costs of HCV testing, liver disease and HCV treatment. We reviewed the literature, and contacted persons involved in the elimination project in the country of Georgia to interpret published data to obtain costs of HCV testing, DAA treatment and liver disease, and HCV- and liver-related utilities (Table 2) (Jülicher & Galli, 2018; Ormeci et al., 2014; Cloherty et al., 2016). We adopted life-time liver disease costs from Turkey for viremic individuals (Ormeci et al., 2014). We assumed that the costs of liver disease in stages F0-F3 decreased by 50% after achieving SVR. The quality of life of patients has been shown to improve substantially after SVR (Dusheiko, 2017). Moreover, the model does not allow liver disease regression, so patients modelled to be in an advanced stage of the liver disease with SVR may in reality have returned to a less severe stage (Knop et al., 2016). It should therefore be safe to assume that the costs of treating liver disease decrease substantially after achieving SVR. In all analyses, we calculated the incremental cost-effectiveness ratios (ICERs) between the strategies, comparing incremental costs with incremental gain in quality-adjusted life expectancy at time of infection. The results are presented per infected individual. We discounted all future costs and quality-adjusted life years (QALYs) at 3% per year.

**Table 2:** Unit costs and health utilities.

Costs	Value	Source
Antibody test	$2	Strategic plan for the elimination of Hepatitis C Virus in Georgia (2020), Chikovani et al. (2019)
Antigen test	$21	
PCR test	$40	
Physician visit and blood collection	$13	
Treatment monitoring costs^a^	$117	Strategic plan for the elimination of Hepatitis C Virus in Georgia (2020)
Treatment cost	$50,674	Scott John (2019)
Average annual cost of liver disease		
Fibrosis stage F0-F2	$447	Ormeci et al. (2014)
Fibrosis stage F3	$447	
Fibrosis stage F4	$578	
Decompensated cirrhosis	$1984	
Hepatocellular carcinoma	$2474	
Health-related utilities		
Fibrosis stage F0-F2	0.82	Knop et al. (2016), Deuffic-Burban et al. (2018)
Fibrosis stage F3	0.76	
Fibrosis stage F4	0.76	
Decompensated cirrhosis	0.60	
HCC	0.60	
F0-F1 after sustained virologic response	0.95	
F2-F4 after sustained virologic response	0.85	

**Notes.**

a Including the cost of clinical assessment, complete blood count, ALT (AST, creatinine), patient service standard. HCC, hepatocellular carcinoma; F0- F4, fibrosis stages according to METAVIR scoring system.

### Sensitivity analyses

We conducted sensitivity analyses to address the uncertainty around key parameters and generalize our findings to other settings (Table S2 ). First, we reduced the unit cost of either the PCR test (sensitivity analysis S1) or antigen test (sensitivity analysis S2). Second, we reduced the liver-related costs after SVR to zero for liver stages F0-F2 (sensitivity analysis S3). Third, we used an alternative estimate of liver disease costs from France (sensitivity analysis S4). Fourth, we calculated the results for a population consisting completely of non-IDUs with decreased HCV prevalence (sensitivity analysis S5), or an IDU population with increased HCV prevalence (sensitivity analysis S6). Finally, we decreased the cost of HCV treatment to generalize the results for settings that have access to treatment with substantially reduced prices (sensitivity analysis S7).

## Results

In the baseline scenario, 89,400 of 100,000 infected individuals were diagnosed during the four-year screening period. In the antibody-antigen strategy, fewer infected individuals were detected (86,100 per 100,000 infected individuals). For antigen test alone the number of diagnoses was 87,500 per 100,000 infected individuals. PCR test alone could detect 91,000 individuals; the remaining 9% of infected individuals either died or spontaneously recovered before testing.

**Figure 2:**
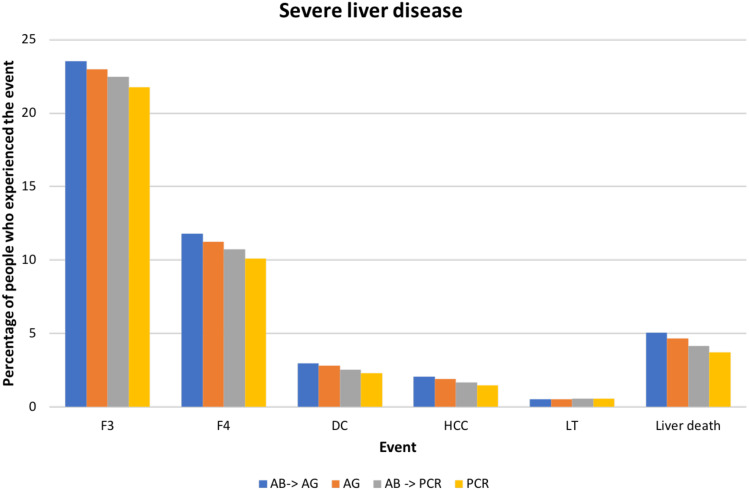
A comparison between different testing strategies: the proportion of infected individuals who experienced different stages of liver disease in their lifetime. F3–F4, stages of fibrosis according to the METAVIR scoring system; DC, decompensated cirrhosis; HCC, hepatocellular carcinoma; LT, Liver transplantation.

The proportion of patients who experienced severe liver disease was highest in the antibody-antigen strategy, and lowest for PCR testing alone (Fig. 2). In the baseline strategy, 22.5% of infected individuals experienced at least liver disease stage F3. The percentages of people who experienced at least liver disease stage F3 were 23.5%, 22.9%, and 21.7% for antibody-antigen, antigen test alone, and PCR test alone, respectively. The percentages of people who reached stage F4 ranged between 10.0% and 12.0% across all strategies. For DC, HCC, LT and liver-related death the corresponding ranges were 2.2%–3.0%, 1.4%–2.1%, 0.5%–0.6% and 3.7%–5.0%, respectively.

**Figure 3:**
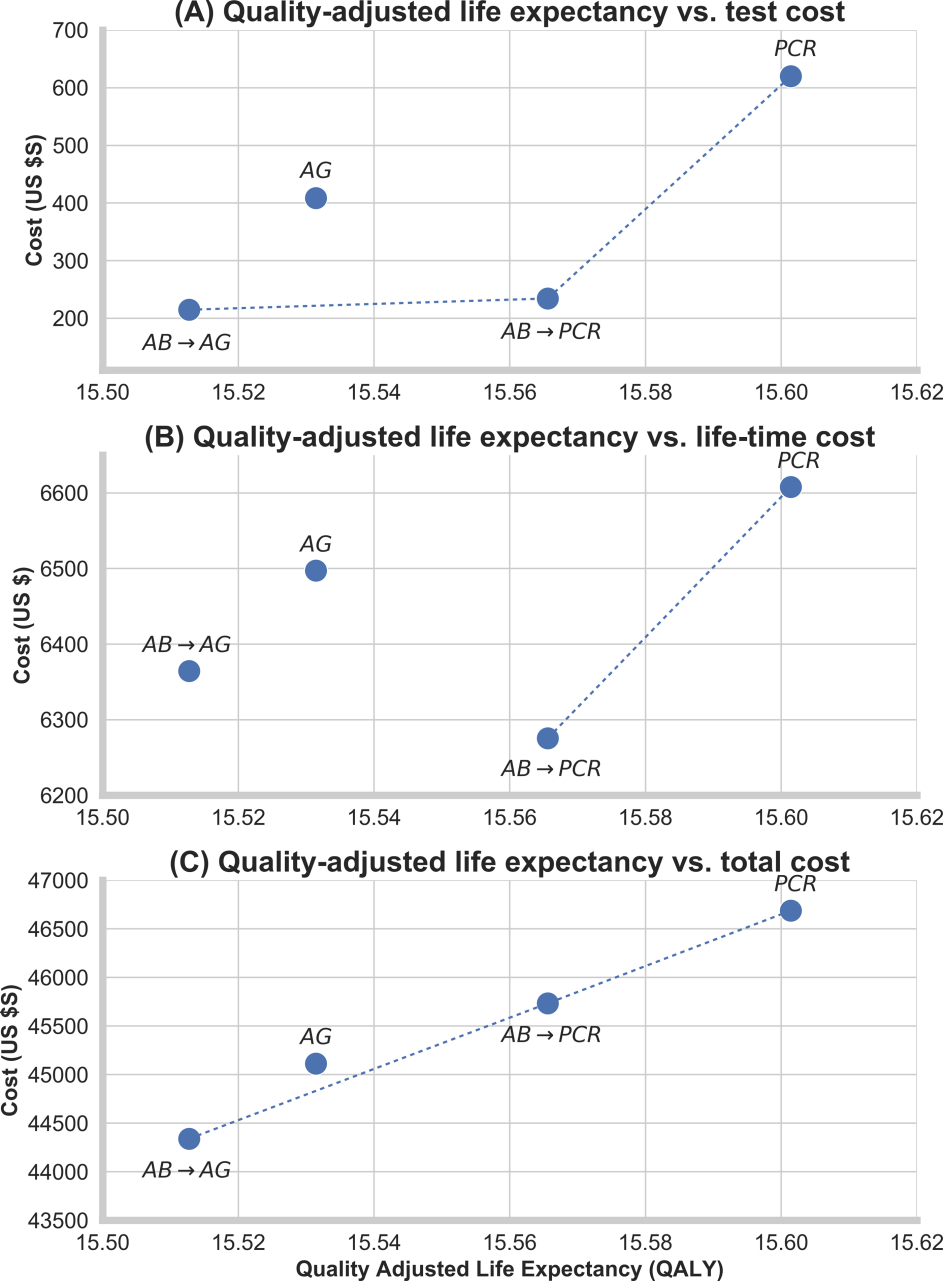
Quality-adjusted life expectancy *versus* cost. (A) Analysis 1: Cost of HCV testing *versus* the quality-adjusted life expectancy. (B) Analysis 2: Cost of HCV testing and life-time liver-related complications *versus* the quality-adjusted life expectancy. (C) Analysis 3: Cost of HCV testing, life-time liver related complications and HCV treatment *versus* the quality-adjusted life expectancy. All costs are measured per infected individual and include also costs of negative tests. Quality-adjusted life expectancy is measured per infected individual at the time of infection. All QALYs and costs are discounted by 3% per year. QALY, quality-adjusted life years; AB, antibody; AG, antigen.

In analysis A1, considering only the cost of testing, antibody-antigen was the cheapest strategy with a total cost of $215 per infected individual and a mean quality-adjusted life expectancy of 15.51 QALYs (Fig. 3A). The most cost-effective strategy compared with antibody followed by antigen was the baseline strategy, with a quality-adjusted life expectancy of 15.56 QALYs and an ICER of $369/QALY gained. Antigen alone had higher costs than the baseline strategy. PCR alone, which had a mean quality-adjusted life expectancy of 15.60 QALYs, was the most effective strategy with an ICER of $10,763/QALY gained compared with the baseline strategy.

In analysis A2 including all costs except treatment (Fig. 3B), the baseline strategy was the cheapest, with a life-time cost of $6,275. PCR test alone, the only strategy performing better than the baseline, had an ICER of $9,281/QALY gained compared with the baseline scenario.

In analysis A3 considering all costs of testing, liver disease and treatment, antibody-antigen was again the cheapest strategy, with an average life-time cost of $35,576 (Fig. 3C). Compared with antibody-antigen strategy, the baseline strategy was the most cost-effective, with an ICER of $19,890/QALY gained. Compared with the baseline, the ICER of PCR testing alone was $22,636/QALY gained.

### Sensitivity analyses

Changing the input costs of diagnostic tests, liver disease or treatment did not change the patterns of cost-effectiveness substantially (Figs. S3–S7). The largest differences were in the analyses of the low- and high-prevalence populations. In a low-prevalence non-IDU population, the results of all three analyses were driven by the costs of testing uninfected individuals. Replacing the two-step testing (antibody-antigen, or baseline strategy) with antigen alone increased the costs of testing (analysis A1) by $2,000, or with PCR alone, by $4,000 per infected individual (Fig. S8). In the high-prevalence IDU population, the situation was reversed (Fig. S9). Considering the costs of testing only (analysis A1), antigen and PCR testing alone were slightly cheaper than their corresponding two-step procedures. If costs of liver disease were also included (analysis A2), PCR testing alone was the cheapest strategy. Considering all costs (analysis A3), antibody-antigen was again the cheapest scenario. Of the remaining strategies, antigen testing alone was most cost-effective with an ICER of $12,265/QALY gained compared to the cheapest strategy.

## Discussion

### Principal findings

Strategies using an antigen test to diagnose HCV infection performed reasonably well compared with the traditional PCR-based approach, but the cost-effectiveness of these strategies depends on the perspective taken. In situations like in the country of Georgia, where treatment is provided from external sources (Ministry of Health of Georgia, 2020), the current two-step testing procedure using antibody and PCR tests has the lowest costs from the healthcare system’s point of view. Adding HCV treatment costs to our analysis made the two-step procedure with confirmation by antigen instead of PCR the cheapest, but also the least effective, strategy. However, the maximum difference in quality-adjusted life expectancy across all strategies was only one month. Antigen testing alone performed better than antibody followed by antigen, but not as well as the baseline strategy. PCR testing alone was clearly the most effective but also most expensive strategy. These additional costs could however be compensated by cost savings related to liver disease, if treatment costs were not considered.

Antigen testing alone is a potential alternative for the current two-step testing procedure. Both strategies miss some HCV infected individuals. In our study, antigen alone missed about 3% and the baseline strategy 2% of those infected. But the characteristics of the missed patients differ. The HCV antibody test can detect an infection only after about 35 days (Ottiger, Gygli & Huber, 2013). Strategies using antibody tests may lead to under-diagnosis in populations with ongoing transmission. In the Georgian HCV epidemic, where most infections were acquired during the first years after the collapse of the Soviet Union (Walker et al., 2018), this may be of limited relevance except for special groups such as IDU. Also, the antibody test may remain negative in immunosuppressed patients (Medici et al., 2011). The antigen test in turn misses around two thirds of individuals with VLVL (Bertisch et al., 2020). Antigen testing was less beneficial than the baseline strategy for two reasons: the number of VLVL patients was higher than the number of recently infected patients; and recently infected patients reach end stage liver disease later than VLVL patient on average. Antigen testing saved costs mainly by missing the individuals with VLVL and therefore reducing the number of treated patients. However, spontaneous cure is more frequent among VLVL individuals than other chronically infected patients (Bertisch et al., 2020), and the probability of onward HCV transmission may also be lower in persons with VLVL (Bouvet, 2005). A one-step simple test could also reduce the risk of loss to follow-up (LTFU). This may be highly relevant for a country like Georgia where, in 2015, more than 25% of anti-HCV positive individuals had no confirmatory testing and were considered LTFU (National Hepatitis C Virus Elimination Progress Report, 2018). A one-step simple test would also reduce the unnecessary anxiety among individuals with a false positive result or spontaneous cure.

Although PCR testing is more expensive than antigen testing, our analysis revealed some situations where the total costs may be lower with PCR based strategies. If treatment costs are not considered, the costs saved by preventing liver disease progression in a few patients could outweigh the additional costs needed for PCR testing. In addition to settings where treatment is covered by external sources, this may also be relevant for countries that have negotiated special agreements with treatment manufacturers. In the “subscription model” (Trusheim, Cassidy & Bach, 2018; Moon & Erickson, 2019), where the government pays a flat fee for treating all infected residents within a given time period, treatment costs do not depend on the number of treated patients and cost-effectiveness evaluations should focus on the costs of liver disease and diagnostics. PCR testing alone was used in our analysis as a theoretical best-case comparator: testing the population with this test costing more than $40 is unlikely. However, in a setting with extremely high prevalence and ongoing transmission, such as active IDU, PCR testing alone could be cost-saving.

When comparing the current practice to the less expensive strategy using antibody followed by antigen testing, the ICER was $19,600 per QALY for all costs including treatment, and the high costs were mainly caused by the increased need of treatment. This shows that in higher-income countries, the use of PCR as the confirmatory test can be justified from the financial point of view. However, in low-income settings, it may not be efficient to invest in PCR tests, in particular if individuals with low viral loads have a lower rate of HCV transmission and higher probability of spontaneous cure. It is notable that in low-income settings, government negotiations and other efforts towards HCV elimination may lead to lower price of DAA treatment and HCV RNA testing, but possibly also antigen testing. The formal cost-effectiveness analysis also does not consider factors such as budget restrictions. With a limited budget, less expensive tests allow to test more individuals, leading to more diagnoses and better clinical outcomes. Under some conditions, the use of antigen as a confirmatory test may thus be beneficial.

### Strengths and limitations

Several studies have compared antigen and PCR testing and proposed the use of antigen testing (Cloherty et al., 2016; Wang, Lv & Zhang, 2017; Alonso et al., 2016). They were however limited to the costs of the diagnosis, or other short-term costs. Our study compares different HCV testing strategies on the life-time burden of HCV infection in a nationwide setting. Our study included the progression of both liver disease and HCV infection. We used different cost perspectives which makes the results of our study applicable for settings with differing financing systems and conditions. We used local programmatic data from Georgia and literature data to parameterize our model.

Our study is subject to limitations. First, HCV transmission was not included; thus, we ignored the additional disease burden and costs that each missed case might cause by onward transmission. Second, we did not consider HCV reinfection after SVR: we assumed that achieving SVR once will lead to a lifetime mitigation of liver related QALY loss and cost. Reinfection will reduce this benefit among people who are correctly tested positive and achieve SVR, which in turn will reduce the overall differences in QALYs and costs between the strategies. Third, we did not model any extrahepatic manifestations (EHM). EHM could increase the overall life-time costs associated with HCV (Younossi et al., 2016), which could further favour more effective testing. Fourth, we did not allow for HCV re-testing. Individuals at high risk of infection, such as active IDUs, are recommended to get retested at least once a year (Gretch, 2000). This is also the population with most acute infections. As antibody testing misses those recently infected, our model may overestimate the benefit of antibody testing for populations with many acute infections. Including re-testing could therefore favour the two-step testing procedures, as the patients who were tested negative during the acute phase of disease using an antibody test could be detected at the next testing round. Fifth, we did not consider some factors favouring the use of antigen testing, such as lower transmission risk and more spontaneous cure among patients with VLVL, and the potentially better retention of one-step testing strategies. Sixth, our analyses took the perspective of a health care payer. In the Georgian elimination program, the care is now free of charge for the patients. In settings where a considerable part of the costs is covered with out-of-pocket payments, the search for the most cost-effective strategy becomes more complicated.

## Conclusions

A single antigen test can be a reliable and practical alternative for the current two-step procedure to diagnose HCV infection, but the cost-effectiveness of this strategy depends on various factors. In settings where the costs of treatment do not directly depend on the number of treated patients, the higher costs of PCR testing are likely compensated by savings in liver disease-related costs. However, a full consideration of treatment-related costs may favour simpler and easier tests such as antigen alone, or antigen after antibody testing. The change to a simple one-time test could offer advantages. In addition, the vast majority of individuals chronically infected with HCV have viral loads above 3,000 IU/ml, a level at which the diagnostic capacity of antigen tests does not differ from PCR. Replacing PCR test by antigen test to diagnose HCV can be considered as a safe option for settings with limited resources, although the associated cost savings may be limited.

## Supplemental Information

10.7717/peerj.11895/supp-1 Supplemental Information 1 Supplemental Figures and Tables

## Additional Information and Declarations

### Data Availability

The following information was supplied regarding data availability: The code is available at GitHub: https://github.com/maryamsadeghimehr/HCV_Antigen.
