# Supplementary material for: Hepatitis C core antigen test as an alternative for diagnosing HCV infection: mathematical model and cost-effectiveness analysis
Source: PeerJ. 2021 Sep 10;9:e11895. doi: 10.7717/peerj.11895 (PMC8436958; doi:10.7717/peerj.11895)
Supplement: Supplemental Information 1 [file peerj-09-11895-s001.docx]

**Supplementary documents**

**Hepatitis C Core Antigen test as an alternative for diagnosing HCV infection: mathematical model and cost-effectiveness analysis**

# **Supplementary Table 1:** Disease progression rates between METAVIR stages F0 and F4 according to gender and current age^1^

| Age | 10-19 | 20-29 | 30-39 | 40-49 | 50-59 | 60-69 | 70-79 | 80+ |
| --- | --- | --- | --- | --- | --- | --- | --- | --- |
| Fibrosis progression rate per 100 person-years: Male | | | | | | | | |
| F0 -> F1 | 4.5 | 3.7 | 2.7 | 9.9 | 12.1 | 13.8 | 15.5 | 12.7 |
| F1 -> F2 | 3.3 | 2.7 | 1.9 | 7.2 | 8.8 | 10.0 | 11.2 | 13.0 |
| F2 -> F3 | 4.7 | 3.8 | 2.8 | 10.2 | 12.4 | 14.1 | 15.9 | 13.0 |
| F3 -> F4 | 0.6 | 1.8 | 4.0 | 6.3 | 3.4 | 7.0 | 13.6 | 13.6 |
| Fibrosis progression rate per 100 person-years: Female | | | | | | | | |
| F0 -> F1 | 3.8 | 3.1 | 2.2 | 8.2 | 10.2 | 11.5 | 12.9 | 10.6 |
| F1 -> F2 | 2.8 | 2.2 | 1.6 | 6.0 | 7.4 | 8.3 | 9.4 | 7.7 |
| F2 -> F3 | 3.9 | 3.1 | 2.3 | 8.5 | 10.4 | 11.8 | 13.2 | 10.9 |
| F3 -> F4 | 0.4 | 1.5 | 3.3 | 5.3 | 2.8 | 5.9 | 11.3 | 11.3 |
| Hazard ratio to modify the progression rate if the patient is a moderate, or excessive alcohol consumer | | | | | | | | |
| F0 -> F1 | 1 non, 1.16 moderate, 1.33 excessive alcohol consumer | | | | | | | |
| F1 -> F2 | 1 non, 1.3 moderate, 2.22excessive alcohol consumer | | | | | | | |
| F2 -> F3 | 1 non, 1.3 moderate, 2.22 excessive alcohol consumer | | | | | | | |
| F3 -> F4 | 1 non, 1.16 moderate, 4 excessive alcohol consumer | | | | | | | |

^1^We used the fibrosis progression rates between METAVIR stages F0 and F4 from a study conducted by Razavi et al. [1] Fibrosis progression rates were back-calculated from data from the US (US Surveillance, Epidemiology and End Results; SEER). They used the results of Harris et al. [2] who used a similar back-calculation method for calculating the fibrosis progression rates for patients from the UK, as a guidance.

**Supplementary Table 2. Sensitivity analyses**

| Sensitivity analysis | Description |
| --- | --- |
| S1 | The cost of PCR test was decreased by 20%. |
| S2 | The cost of antigen test was decreased by 30%. |
| S3 | Change the cost of liver disease after SVR to zero. |
| S4 | Different set of liver disease costs (based on data from France)^[4]^ . |
| S5 | Low prevalence (0.7%) among non-IDU (looking at only non-IDUs cohort). |
| S6 | High prevalence (94%) among IDU (looking at only IDUs cohort). |
| S7 | The cost of treatment was reduced to $44. |

IDU = Intravenous Drug Users

# **Supplementary Fig. 1. Distribution of HCV viral load in the simulated cohort.** The y-axis shows the percentage of the simulated individuals for each HCV viral load level.


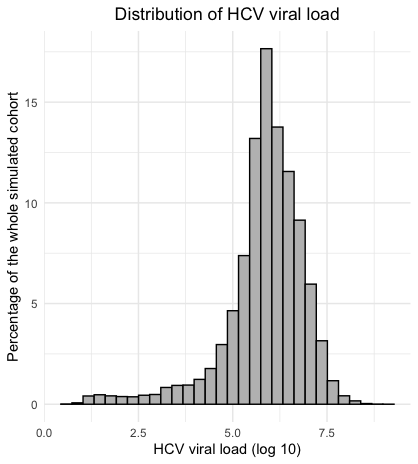


**Supplementary Fig. 2. Distribution of age and year of HCV infection in the simulated cohort.**  The total area shows the percentage of the simulated cohort (patients who were viremic and alive between 2015 and 2018) infected each year, and the colors the distribution of age at time of infection. This distribution was determined to obtain the distribution of age at diagnosis based on a study of Turdziladze et al. [3].


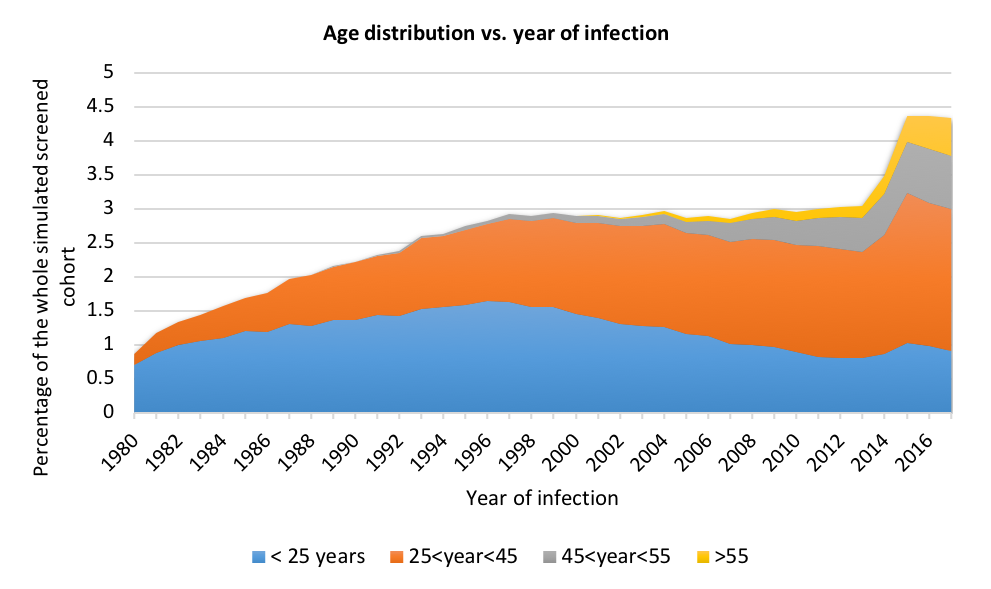


**Supplementary Fig. 3. Sensitivity analyses of laboratory test costs (S1): Cost of PCR test was decreased by 20%.** Antibody→PCR, antibody test followed by PCR, Antibody→Antigen: antibody test followed by antigen test. AB: antibody; AG: antigen.

**
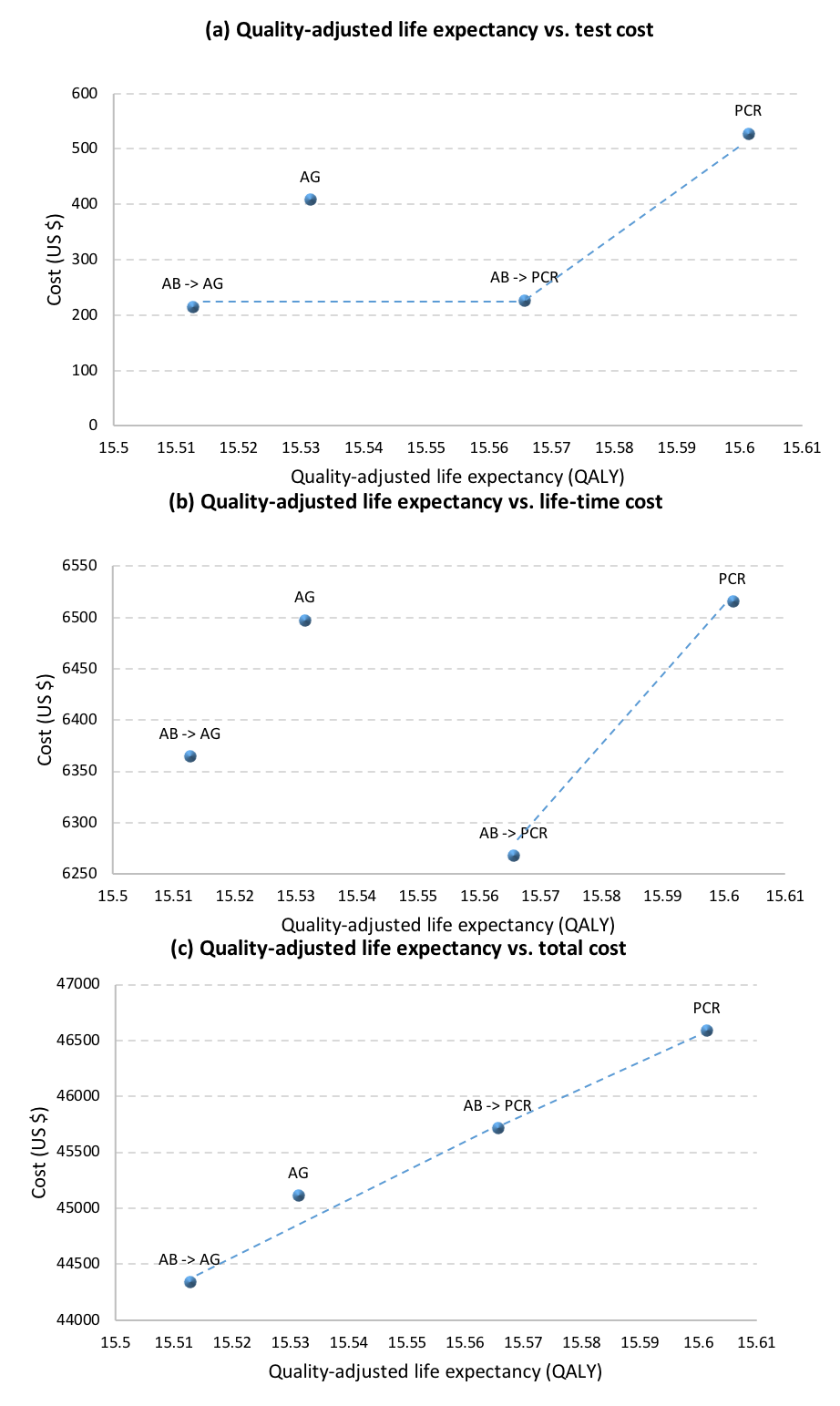
**

**Supplementary Fig. 4. Sensitivity analyses of laboratory test costs (S2): Cost of HCV antigen test decreased by 30%.** Antibody→PCR, antibody test followed by PCR, Antibody→Antigen: antibody test followed by antigen test. *AB: antibody; AG: antigen.*

**
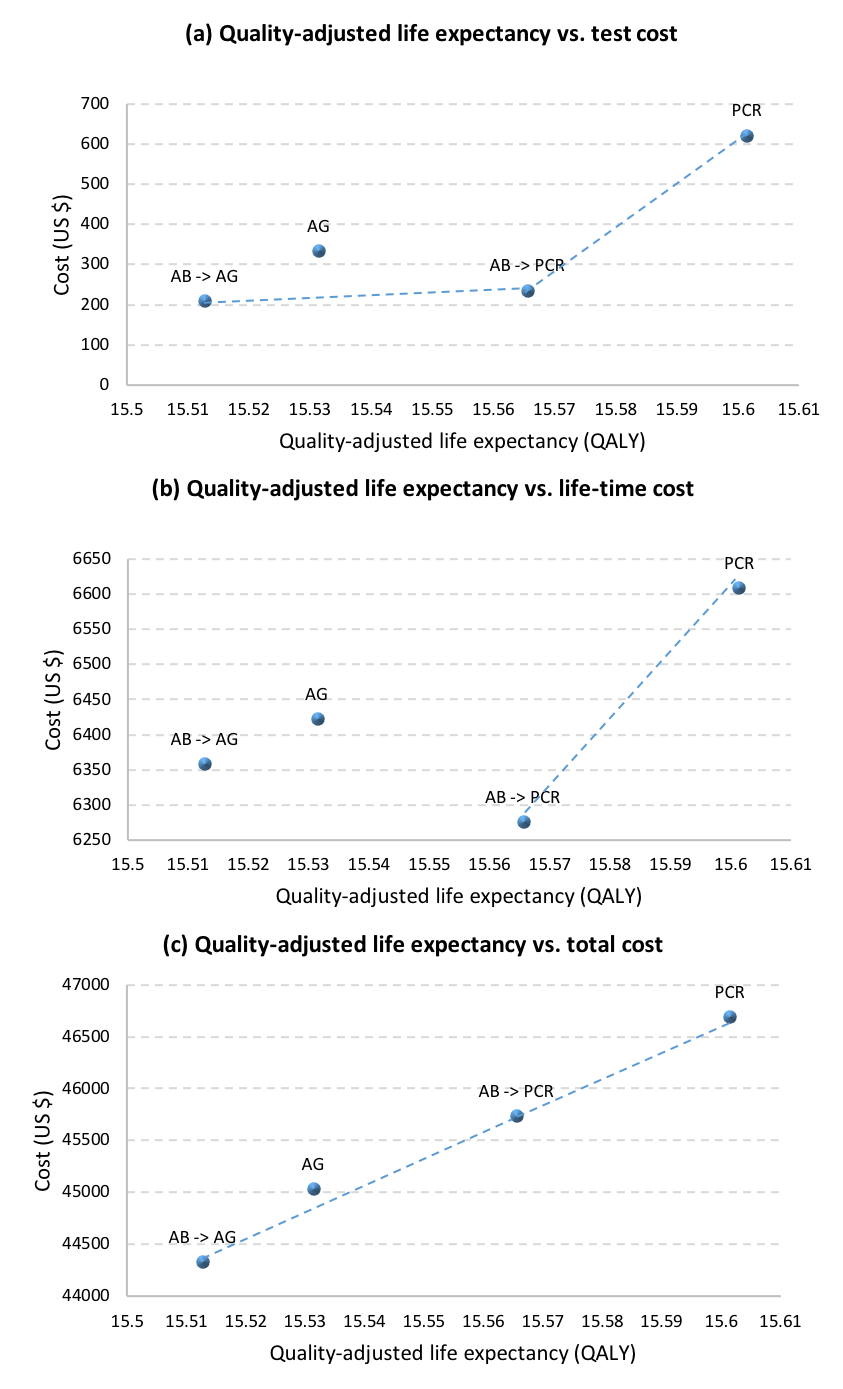
**

**Supplementary Fig. 5. Sensitivity analyses of annual liver-related costs attributable to chronic hepatitis C costs (S3): Change the cost of liver disease after SVR to zero.** Antibody→PCR, antibody test followed by PCR, Antibody→Antigen: antibody test followed by antigen test. AB: antibody; AG: antigen.

# **
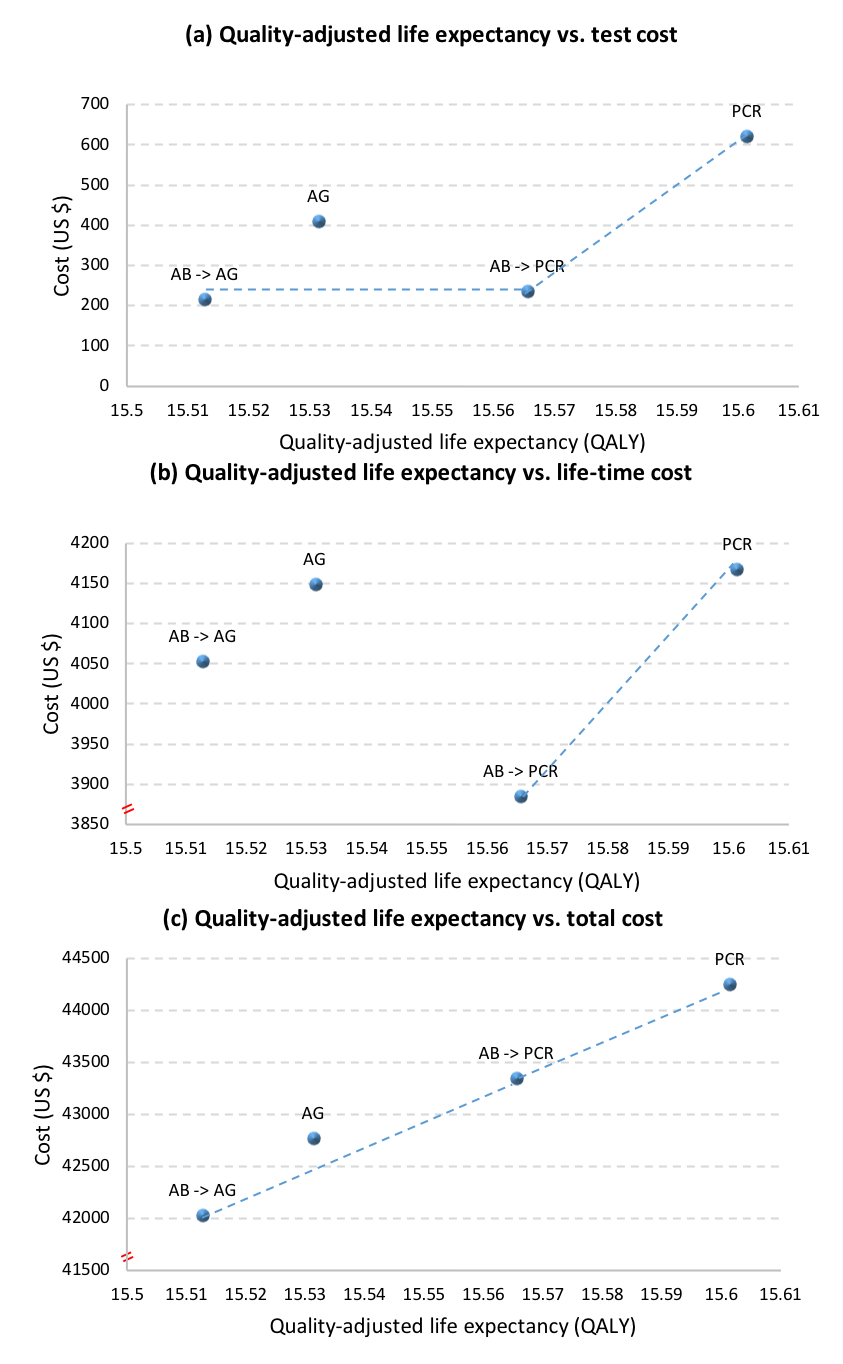
**

# **Supplementary Fig. 6. Sensitivity analyses of annual liver-related costs attributable to chronic hepatitis C costs (S4): Different set of liver disease costs (based on data from France).** Antibody→PCR, antibody test followed by PCR, Antibody→Antigen: antibody test followed by antigen test. *AB: antibody; AG: antigen.*


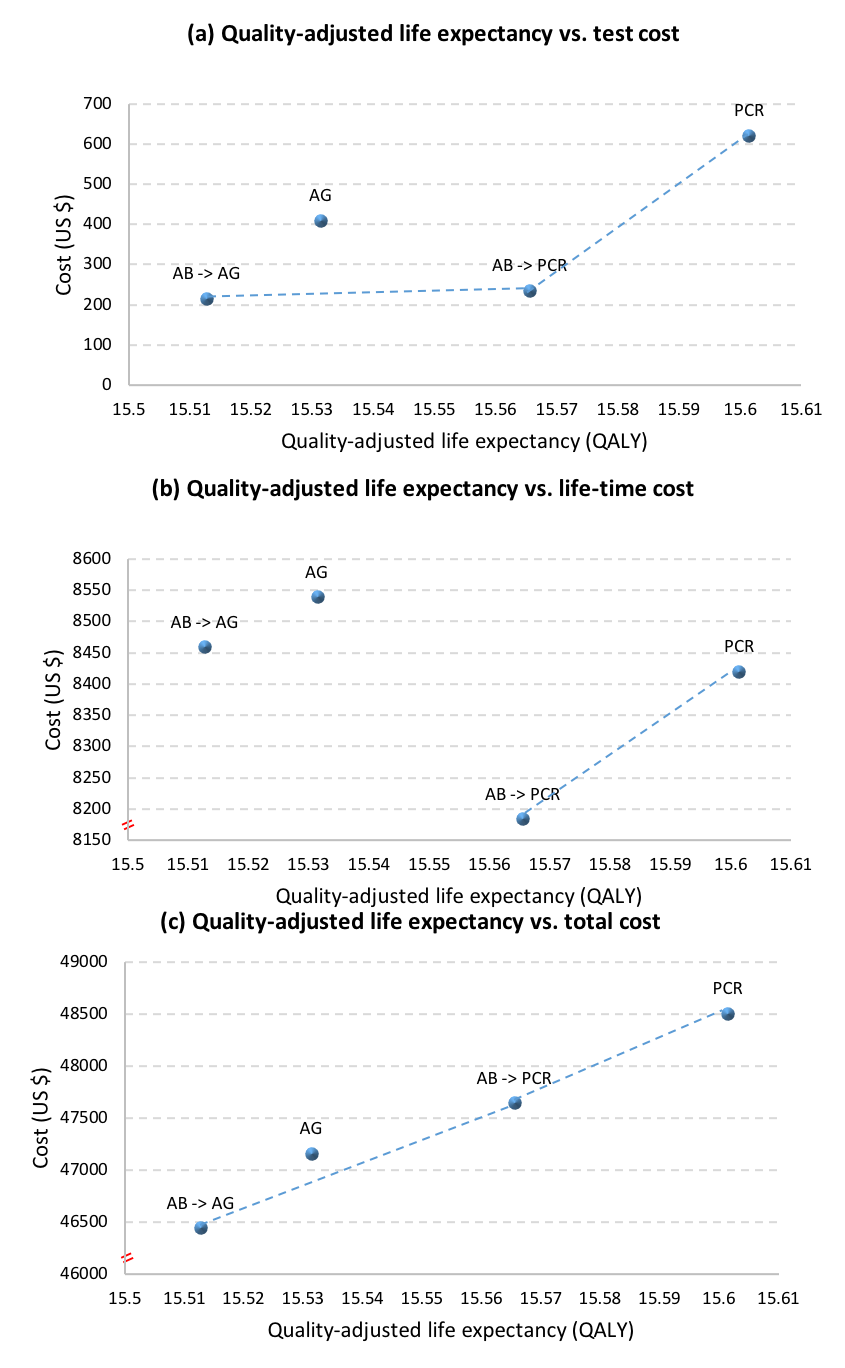


# **Supplementary Fig. 7. Substantially reducing the cost of HCV treatment ((S7)).** Antibody→PCR, antibody test followed by PCR, Antibody→Antigen: antibody test followed by antigen test. *AB: antibody; AG: antigen.*


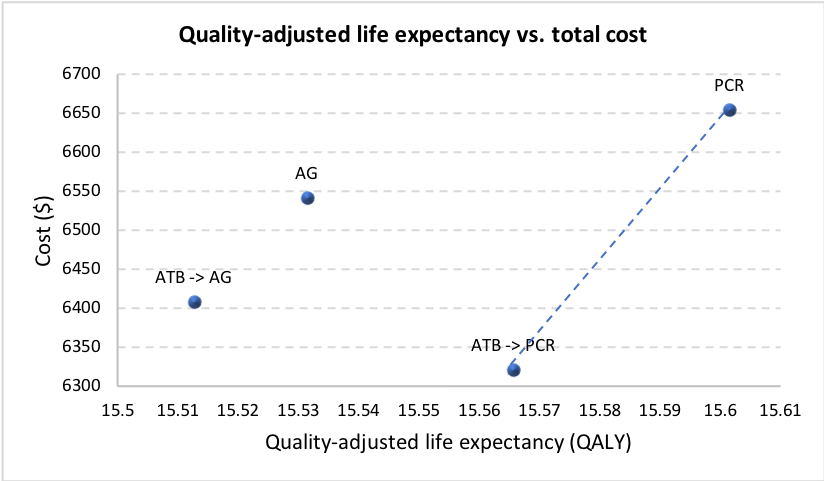


**Supplementary Fig. 8. Sensitivity analyses of prevalence (low prevalence among non-IDU population (S5)).** Antibody→PCR, antibody test followed by PCR, Antibody→Antigen: antibody test followed by antigen test. *AB: antibody; AG: antigen.*


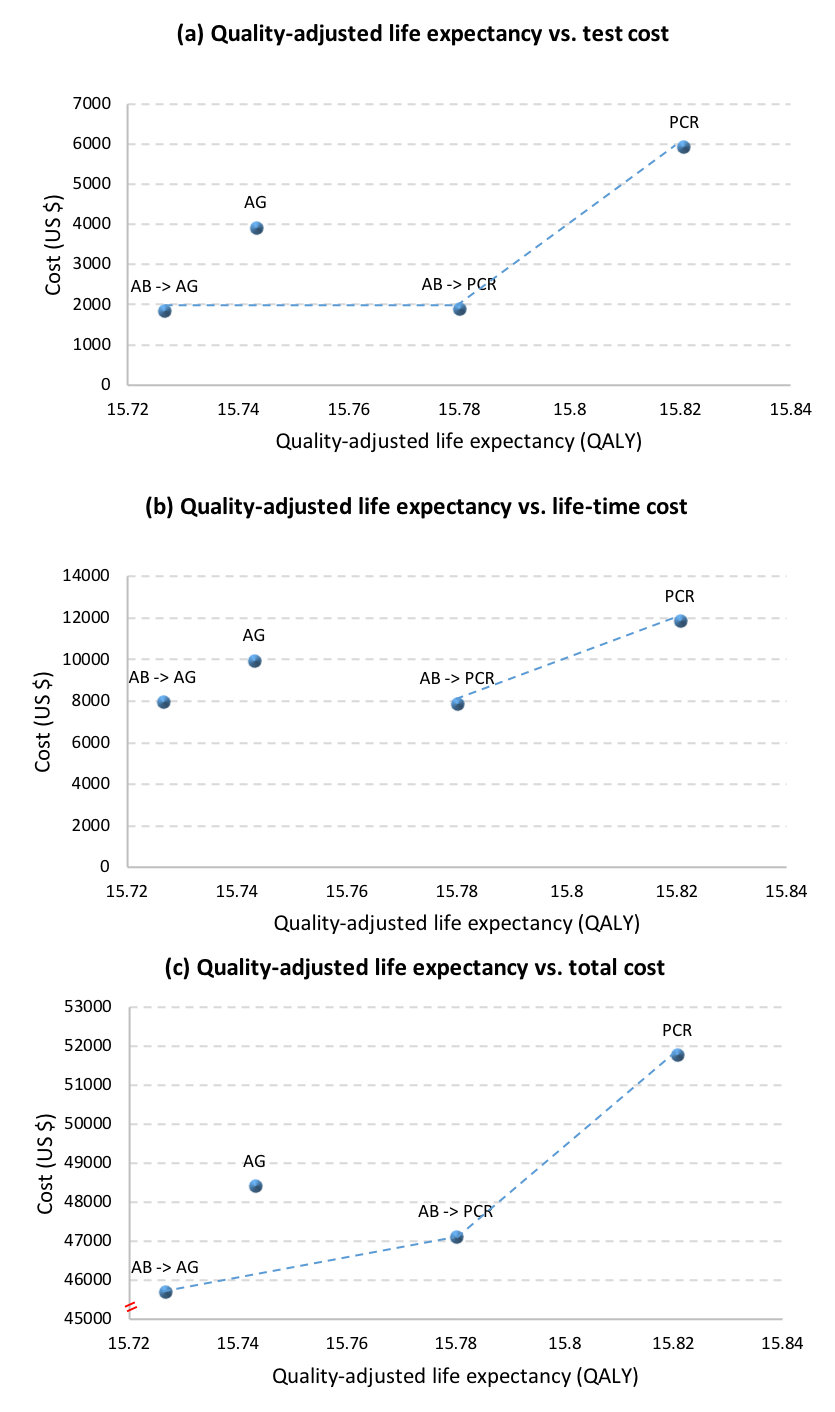


**Supplementary Fig. 9. Sensitivity analyses of prevalence (high-prevalence among IDUs population (S6)).** Antibody→PCR, antibody test followed by PCR, Antibody→Antigen: antibody test followed by antigen test. *AB: antibody; AG: antigen.*


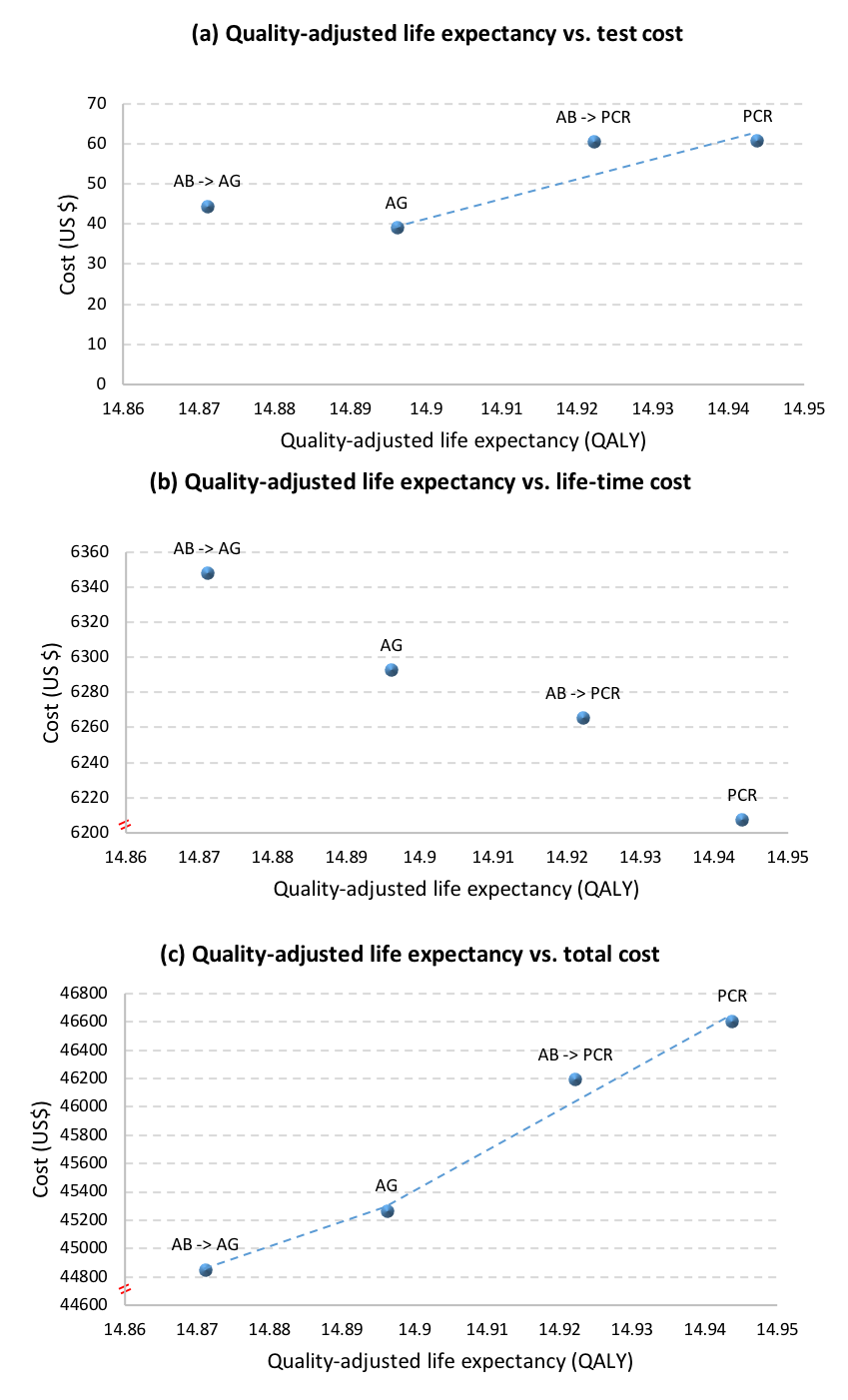


**References**

[1] Razavi H, WakedI, Sarrazin C*et al.* The present and future disease burden of hepatitis C virus (HCV) infection with today’s treatment paradigm. *J Viral Hepat* 2014; **21** (**s1**): 34–59.

[2] Harris RJ, Thomas B, Griffiths J *et al.* Increased uptake and new therapies are needed to avert rising hepatitis C-related end stage liver disease in England: modelling the predicted impact of treatment under different scenarios. *J Hepatol* 2014; **61**: 530–537.

[3] Gamkrelidze A, Turdziladze A, Getia V, Alkhazashvili M, Tsereteli M, Aslanikashvili A, Baliashvili D, Imnadze P. Hepatitis C screening within the national elimination program in the country of Georgia. *J Hepatol*. 2018;68:S158. doi:10.1016/s0168-8278(18)30528-2.

[4] Deuffic-Burban S, Huneau A, Verleene A, et al. Assessing the cost-effectiveness of hepatitis C screening strategies in France. *J Hepatol*. 2018;69(4):785-792. doi:10.1016/j.jhep.2018.05.027
